# Secohellebrigeninamide

**DOI:** 10.1107/S160053681200520X

**Published:** 2012-02-10

**Authors:** Xiao-Feng Yuan, Hai-Yan Tian, Jin-Hang Li, Tong Yu, Ren-Wang Jiang

**Affiliations:** a Guangdong Province Key Laboratory of Pharmacodynamic Constituents of Traditional Chinese Medicine and New Drugs Research, Institute of Traditional Chinese Medicine and Natural Products, Jinan University, Guangzhou 510632, People’s Republic of China

## Abstract

The title compound, C_26_H_37_NO_5_, was the reaction product of hellebrigenin with *N*,*N*-dimethyl­formamide. It consists of three cyclo­hexane rings (*A*, *B* and *C*), one five-membered ring (*D*) and one dihydro­pyran ring (*E*). The stereochemistry of the ring junctions is is *A*/*B cis*, *B*/*C trans*, *C*/*D cis* and *C*/*E trans*. The cyclo­hexane rings *A*, *B* and *C* have chair conformations. Both the five-membered ring *D* and the dihydro­pyran ring adopt an envelope conformation. Two orientations are found for the aldehyde group with occupancies of 0.608 (10) and 0.392 (10). In the crystal, short O—H⋯O hydrogen bonds and short C—H⋯O contacts involving the hy­droxy group, terminal methyl group and carbonyl group link the mol­ecules into a three-dimensional network.

## Related literature

For previous isolation of hellebrigenin, see: Urscheler *et al.* (1955 ▶); Yang *et al.* (2010 ▶); Zhao *et al.* (2010 ▶). For the treatment of hellebrigenin with sodium hydroxide, see: Kupchan *et al.* (1969 ▶). For the stereochemistry of bufalin, see: Rohrer *et al.* (1982 ▶).
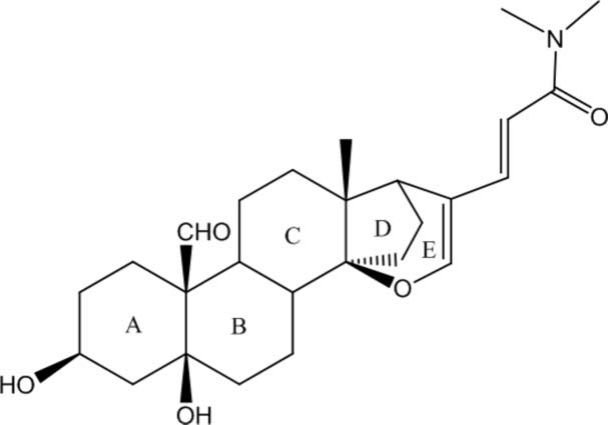


## Experimental

### 

#### Crystal data

C_26_H_37_NO_5_
*M* *_r_* = 443.57
Monoclinic, 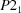

*a* = 6.6942 (1) Å
*b* = 16.0580 (4) Å
*c* = 10.9672 (3) Å
β = 98.693 (2)°
*V* = 1165.38 (5) Å^3^
*Z* = 2
Cu *K*α radiation
μ = 0.70 mm^−1^
*T* = 291 K
0.38 × 0.30 × 0.25 mm

#### Data collection

Oxford Diffraction Gemini S Ultra Sapphire CCD diffractometer
Absorption correction: multi-scan (*SADABS*; Sheldrick, 2004 ▶) *T*_min_ = 0.673, *T*_max_ = 1.000
3575 measured reflections
2534 independent reflections
2431 reflections with *I* > 2σ(*I*)
*R*_int_ = 0.021

#### Refinement

*R*[*F* ^2^ > 2σ(*F* ^2^)] = 0.046
*wR*(*F* ^2^) = 0.130
*S* = 1.04
2534 reflections
293 parameters
1 restraint
H-atom parameters constrained
Δρ_max_ = 0.18 e Å^−3^
Δρ_min_ = −0.16 e Å^−3^

### 

Data collection: *CrysAlis PRO* (Agilent, 2011 ▶); cell refinement: *CrysAlis PRO*; data reduction: *XPREP* in *SHELXTL* (Sheldrick, 2008 ▶); program(s) used to solve structure: *SHELXTL*; program(s) used to refine structure: *SHELXTL*; molecular graphics: *XP* in *SHELXTL*; software used to prepare material for publication: *SHELXTL*.

## Supplementary Material

Crystal structure: contains datablock(s) I, global. DOI: 10.1107/S160053681200520X/vm2153sup1.cif

Structure factors: contains datablock(s) I. DOI: 10.1107/S160053681200520X/vm2153Isup2.hkl

Additional supplementary materials:  crystallographic information; 3D view; checkCIF report

## Figures and Tables

**Table 1:** Hydrogen-bond geometry (Å, °)

*D*—H⋯*A*	*D*—H	H⋯*A*	*D*⋯*A*	*D*—H⋯*A*
O1—H1*A*⋯O5^i^	0.82	1.97	2.790 (3)	178
C25—H25*C*⋯O1^ii^	0.96	2.64	3.513 (3)	152

Symmetry codes: (i) 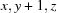; (ii) 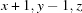.

**Fig. 1.**
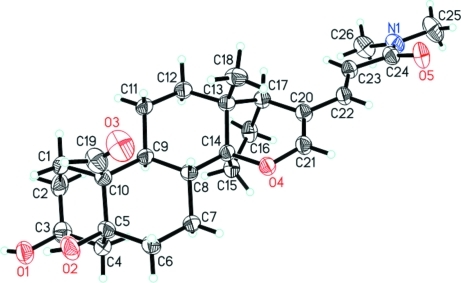
Molecular structure of the title compound showing 30% probability displacement ellipsoids. Only one orientation of the aldehyde group is shown.

**Fig. 2.**
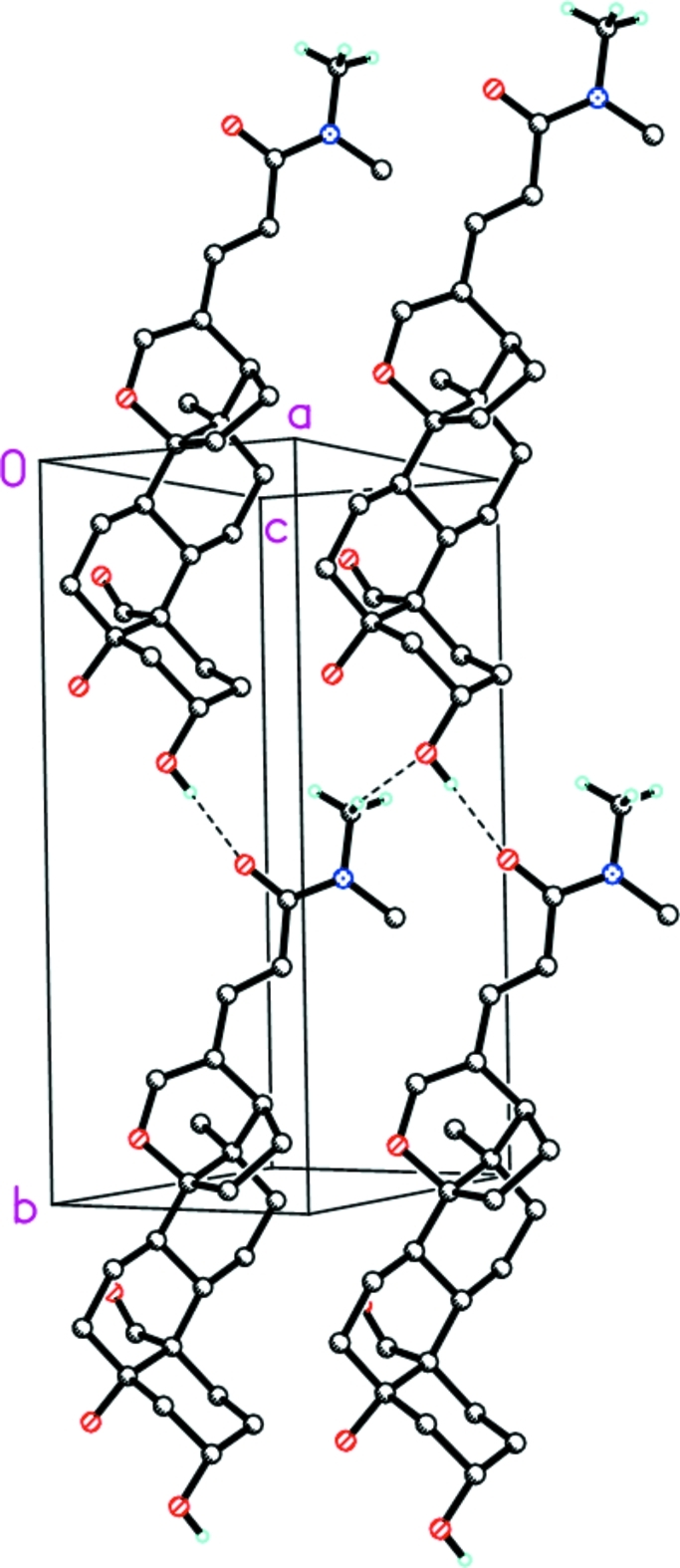
Packing diagram showing the intermolecular O—H···O hydrogen bonds and short C—H···O contacts represented as dashed lines. Only H-atoms involved in interactions are shown.

## supplementary crystallographic information

### Comment

Hellebrigenin is a cardiac steroid. It was firstly isolated from the European toad in 1955 (Urscheler *et al.*, 1955). Since then, it was isolated from the rhizomes of Helleborus thibetanus (Yang *et al.*, 2010) and the skin of the Chinese toad Bufo bufo gargarizans (Zhao *et al.*, 2010). The lactone ring at C-17 is not stable in alkaline conditions. Treatment of hellebrigenin with sodium hydroxide in methanol affords methyl isohellebrigeninate (Kupchan *et al.*, 1969). Recently we treated hellebrigenin with *N*,*N*-dimethylformamide (DMF), and a new derivative named secohellebrigeninamide was obtained. We report herein the crystal structure of this compound.

The colorless blocks of crystals were obtained by recrystallization from the methanol solution at room temperature. The molecule (Fig. 1) is composed of three cyclohexane rings (A, B and C), one five-membered ring (D) and one dihydropyran ring (E). The stereochemistry of the ring juncture is A/B *cis*, B/C *trans*, C/D *cis* and C/E *trans*.

The cyclohexane rings A, B and C have normal chair conformations. The five-membered ring D adopts an envelope conformation with C13 displaced by 0.7246 (2) Å from the mean plane of the remaining four atoms (C14, C15, C16 and C17). Similarly, the dihydropyran ring E also adopts an envelope conformation with C13 displaced by 0.8848 (3) Å from the mean plane of the remaining five atoms (C14, C17, C20, C21 and O4). The absolute configuration determined for bufalin (Rohrer *et al.*, 1982), a similar cardiac steroid, was invoked, giving the assignments of the chiral centres in the molecule as shown in Fig. 1.

A short intermolecular O—H···O hydrogen bond (Table 1) between the hydroxyl group at C3 and the carbonyl group at C24 [O1—H1A···O5^i^, 2.790 (4) Å; symmetry code: (i) *x*,*y* + 1, *z*] links adjacent molecules into chains along the *b*-axis. Adjacent chains are linked by short C—H···O contacts between the terminal methyl group and the hydroxyl group at C3 [C25—H25C···O1^ii^, 3.513 (3) Å; symmetry code: (ii) *x* + 1, *y* - 1, *z*] into a three-dimensional network (Fig. 2).

### Experimental

Hellebrigenin (41.6 mg) was dissolved in DMF and refluxed for 3 h. After the reaction, the mixture was poured into water and extracted with ethyl acetate. The ethyl acetate extract was washed with water to remove the remaining DMF and condensed by a rotary evaporator under reduced pressure. The residue was recrystallized in methanol at room temperature to afford colorless crystals (32.5 mg) suitable for X-ray analysis.

### Refinement

The C-bound H atoms were positioned geometrically and were included in the refinement in the riding-model approximation, with C—H = 0.96 Å (CH_3_) and *U*_iso_(H) = 1.5*U*_eq_(C); 0.97 Å (CH_2_) and *U*_iso_(H) = 1.2*U*_eq_(C); 0.98 Å (CH) and *U*_iso_(H) = 1.2*U*_eq_(C); 0.93 Å (aryl H) and *U*_iso_(H)= 1.2*U*_eq_(C); O—H = 0.82 Å and *U*_iso_(H) = 1.5*U*_eq_(O). O3 and H19 are disordered over two positions with occupancies of 0.608 (10) and 0.392 (10). The Friedel pair coverage for the collection is low. It may be due to an inadequate collection strategy. Recollection of diffraction data was not thought to be necessary since the absolute configuration can be unambiguously assigned with reference to the known configuration of the closed related compound bufalin. The absolute structure is indeterminate.

### Crystal data

**Table:**

C_26_H_37_NO_5_	*F*(000) = 480
*M**_r_* = 443.57	*D*_x_ = 1.264 Mg m^−^^3^
Monoclinic, *P*2_1_	Cu *K*α radiation, λ = 1.54184 Å
*a* = 6.6942 (1) Å	Cell parameters from 1847 reflections
*b* = 16.0580 (4) Å	θ = 6.2–62.6°
*c* = 10.9672 (3) Å	µ = 0.70 mm^−^^1^
β = 98.693 (2)°	*T* = 291 K
*V* = 1165.38 (5) Å^3^	Block, colorless
*Z* = 2	0.38 × 0.30 × 0.25 mm

### Data collection

**Table:**

Oxford Diffraction Gemini S Ultra Sapphire CCD diffractometer	2534 independent reflections
Radiation source: fine-focus sealed tube	2431 reflections with *I* > 2σ(*I*)
Graphite monochromator	*R*_int_ = 0.021
ω scan	θ_max_ = 62.7°, θ_min_ = 5.5°
Absorption correction: multi-scan (*SADABS*; Sheldrick, 2004)	*h* = −2→7
*T*_min_ = 0.673, *T*_max_ = 1.000	*k* = −18→16
3575 measured reflections	*l* = −12→11

### Refinement

**Table:**

Refinement on *F*^2^	Primary atom site location: structure-invariant direct methods
Least-squares matrix: full	Secondary atom site location: difference Fourier map
*R*[*F*^2^ > 2σ(*F*^2^)] = 0.046	Hydrogen site location: inferred from neighbouring sites
*wR*(*F*^2^) = 0.130	H-atom parameters constrained
*S* = 1.04	*w* = 1/[σ^2^(*F*_o_^2^) + (0.0899*P*)^2^ + 0.1076*P*] where *P* = (*F*_o_^2^ + 2*F*_c_^2^)/3
2534 reflections	(Δ/σ)_max_ < 0.001
293 parameters	Δρ_max_ = 0.18 e Å^−^^3^
1 restraint	Δρ_min_ = −0.16 e Å^−^^3^

### Special details

**Table:**

Geometry. All e.s.d.'s (except the e.s.d. in the dihedral angle between two l.s. planes) are estimated using the full covariance matrix. The cell e.s.d.'s are taken into account individually in the estimation of e.s.d.'s in distances, angles and torsion angles; correlations between e.s.d.'s in cell parameters are only used when they are defined by crystal symmetry. An approximate (isotropic) treatment of cell e.s.d.'s is used for estimating e.s.d.'s involving l.s. planes.
Refinement. Refinement of *F*^2^ against ALL reflections. The weighted *R*-factor *wR* and goodness of fit *S* are based on *F*^2^, conventional *R*-factors *R* are based on *F*, with *F* set to zero for negative *F*^2^. The threshold expression of *F*^2^ > σ(*F*^2^) is used only for calculating *R*-factors(gt) *etc*. and is not relevant to the choice of reflections for refinement. *R*-factors based on *F*^2^ are statistically about twice as large as those based on *F*, and *R*- factors based on ALL data will be even larger.

### Fractional atomic coordinates and isotropic or equivalent isotropic displacement parameters (Å^2^)

**Table:**

	*x*	*y*	*z*	*U*_iso_*/*U*_eq_	Occ. (<1)
O1	0.3441 (4)	1.40909 (16)	0.1536 (2)	0.0750 (6)	
H1A	0.4127	1.4495	0.1793	0.113*	
O2	0.0185 (3)	1.30160 (16)	0.1275 (2)	0.0737 (7)	
H2A	0.0758	1.3465	0.1413	0.111*	
O3	−0.0767 (8)	1.1408 (4)	0.3299 (6)	0.1087 (18)	0.608 (10)
O3A	−0.0065 (11)	1.2140 (6)	0.4159 (9)	0.1087 (18)	0.39
O4	0.2368 (3)	0.91532 (13)	0.12249 (17)	0.0530 (5)	
O5	0.5706 (4)	0.54857 (15)	0.2410 (3)	0.0815 (7)	
N1	0.9026 (4)	0.56933 (19)	0.3016 (3)	0.0666 (7)	
C1	0.3354 (5)	1.2767 (2)	0.3439 (3)	0.0618 (8)	
H1B	0.2441	1.3234	0.3468	0.074*	
H1C	0.3765	1.2577	0.4279	0.074*	
C2	0.5202 (5)	1.3069 (3)	0.2931 (3)	0.0712 (9)	
H2B	0.5820	1.3521	0.3440	0.085*	
H2C	0.6177	1.2619	0.2968	0.085*	
C3	0.4688 (5)	1.3362 (2)	0.1624 (3)	0.0674 (9)	
H3A	0.5939	1.3489	0.1298	0.081*	
C4	0.3537 (5)	1.2682 (2)	0.0838 (3)	0.0634 (8)	
H4A	0.3137	1.2896	0.0010	0.076*	
H4B	0.4445	1.2217	0.0784	0.076*	
C5	0.1669 (4)	1.2363 (2)	0.1317 (3)	0.0560 (7)	
C6	0.0671 (5)	1.1673 (2)	0.0493 (3)	0.0671 (9)	
H6A	−0.0642	1.1554	0.0724	0.080*	
H6B	0.0456	1.1868	−0.0354	0.080*	
C7	0.1889 (6)	1.0873 (2)	0.0561 (3)	0.0644 (8)	
H7A	0.3133	1.0969	0.0231	0.077*	
H7B	0.1125	1.0450	0.0058	0.077*	
C8	0.2392 (4)	1.05563 (19)	0.1893 (2)	0.0473 (6)	
H8A	0.1111	1.0420	0.2177	0.057*	
C9	0.3438 (4)	1.12326 (18)	0.2759 (2)	0.0483 (6)	
H9A	0.4721	1.1361	0.2472	0.058*	
C10	0.2204 (4)	1.2060 (2)	0.2691 (2)	0.0515 (7)	
C11	0.3965 (5)	1.0893 (2)	0.4077 (2)	0.0599 (8)	
H11A	0.4701	1.1314	0.4598	0.072*	
H11B	0.2728	1.0772	0.4403	0.072*	
C12	0.5241 (5)	1.0104 (2)	0.4113 (3)	0.0613 (8)	
H12A	0.5511	0.9903	0.4955	0.074*	
H12B	0.6528	1.0240	0.3857	0.074*	
C13	0.4233 (4)	0.94111 (19)	0.3285 (2)	0.0497 (6)	
C14	0.3650 (4)	0.97681 (19)	0.1972 (2)	0.0445 (6)	
C15	0.5674 (4)	0.9822 (2)	0.1456 (3)	0.0545 (7)	
H15A	0.5486	0.9665	0.0592	0.065*	
H15B	0.6208	1.0384	0.1535	0.065*	
C16	0.7123 (4)	0.9212 (2)	0.2229 (3)	0.0598 (8)	
H16A	0.8179	0.9508	0.2760	0.072*	
H16B	0.7739	0.8835	0.1704	0.072*	
C17	0.5736 (4)	0.8739 (2)	0.2993 (3)	0.0520 (6)	
H17A	0.6491	0.8492	0.3741	0.062*	
C18	0.2473 (5)	0.9056 (3)	0.3841 (3)	0.0682 (9)	
H18A	0.1603	0.9501	0.4018	0.102*	
H18B	0.2976	0.8766	0.4590	0.102*	
H18C	0.1727	0.8677	0.3267	0.102*	
C19	0.0265 (6)	1.1947 (3)	0.3181 (4)	0.0756 (10)	
H19A	−0.0214	1.2446	0.3458	0.091*	0.608 (10)
H19B	−0.0789	1.1699	0.2657	0.091*	0.392 (10)
C20	0.4571 (4)	0.8096 (2)	0.2173 (3)	0.0509 (7)	
C21	0.2995 (4)	0.83593 (19)	0.1362 (3)	0.0533 (7)	
H21A	0.2281	0.7961	0.0855	0.064*	
C22	0.5116 (4)	0.7228 (2)	0.2182 (3)	0.0519 (7)	
H22A	0.4238	0.6878	0.1682	0.062*	
C23	0.6737 (4)	0.6871 (2)	0.2826 (3)	0.0533 (7)	
H23A	0.7655	0.7195	0.3343	0.064*	
C24	0.7095 (5)	0.5968 (2)	0.2728 (3)	0.0584 (7)	
C25	0.9418 (7)	0.4814 (3)	0.2967 (6)	0.1014 (15)	
H25A	0.8161	0.4518	0.2801	0.152*	
H25B	1.0148	0.4634	0.3744	0.152*	
H25C	1.0208	0.4703	0.2324	0.152*	
C26	1.0793 (5)	0.6229 (3)	0.3278 (4)	0.0850 (12)	
H26A	1.0369	0.6799	0.3291	0.127*	
H26B	1.1632	0.6156	0.2649	0.127*	
H26C	1.1543	0.6084	0.4065	0.127*	

### Atomic displacement parameters (Å^2^)

**Table:**

	*U*^11^	*U*^22^	*U*^33^	*U*^12^	*U*^13^	*U*^23^
O1	0.0879 (15)	0.0492 (13)	0.0877 (15)	−0.0031 (12)	0.0124 (12)	−0.0062 (12)
O2	0.0705 (12)	0.0510 (13)	0.0906 (16)	0.0170 (12)	−0.0169 (11)	−0.0032 (13)
O3	0.091 (3)	0.098 (4)	0.147 (4)	0.003 (3)	0.050 (3)	0.008 (3)
O3A	0.091 (3)	0.098 (4)	0.147 (4)	0.003 (3)	0.050 (3)	0.008 (3)
O4	0.0530 (10)	0.0431 (11)	0.0563 (10)	−0.0014 (9)	−0.0130 (8)	−0.0010 (9)
O5	0.0729 (14)	0.0478 (14)	0.122 (2)	−0.0068 (13)	0.0100 (13)	−0.0097 (15)
N1	0.0637 (15)	0.0555 (16)	0.0823 (17)	0.0165 (13)	0.0171 (12)	0.0104 (14)
C1	0.0785 (19)	0.0551 (19)	0.0496 (14)	0.0123 (15)	0.0023 (13)	−0.0106 (14)
C2	0.0645 (17)	0.062 (2)	0.080 (2)	−0.0001 (17)	−0.0106 (15)	−0.0223 (19)
C3	0.0655 (17)	0.060 (2)	0.079 (2)	−0.0063 (16)	0.0204 (15)	−0.0040 (17)
C4	0.085 (2)	0.0552 (18)	0.0507 (14)	0.0045 (16)	0.0126 (13)	−0.0032 (14)
C5	0.0621 (15)	0.0452 (16)	0.0545 (15)	0.0085 (14)	−0.0114 (12)	0.0027 (14)
C6	0.079 (2)	0.0498 (19)	0.0622 (17)	0.0052 (17)	−0.0229 (15)	0.0032 (16)
C7	0.088 (2)	0.0491 (18)	0.0469 (14)	0.0056 (16)	−0.0193 (13)	−0.0018 (14)
C8	0.0463 (12)	0.0452 (15)	0.0460 (13)	0.0063 (12)	−0.0065 (10)	0.0009 (12)
C9	0.0524 (13)	0.0479 (16)	0.0415 (13)	0.0067 (12)	−0.0027 (10)	−0.0028 (12)
C10	0.0534 (14)	0.0478 (16)	0.0516 (14)	0.0081 (13)	0.0027 (11)	0.0007 (13)
C11	0.0778 (18)	0.0543 (18)	0.0423 (13)	0.0158 (16)	−0.0083 (12)	−0.0046 (13)
C12	0.0705 (17)	0.0562 (19)	0.0494 (14)	0.0096 (16)	−0.0157 (13)	−0.0019 (14)
C13	0.0492 (13)	0.0487 (16)	0.0463 (13)	0.0076 (13)	−0.0091 (10)	0.0006 (12)
C14	0.0426 (12)	0.0424 (14)	0.0451 (13)	−0.0002 (12)	−0.0041 (10)	−0.0032 (11)
C15	0.0557 (14)	0.0477 (16)	0.0611 (15)	−0.0030 (14)	0.0123 (12)	−0.0072 (14)
C16	0.0442 (13)	0.0497 (18)	0.0839 (19)	−0.0017 (13)	0.0047 (12)	−0.0115 (16)
C17	0.0474 (12)	0.0474 (15)	0.0558 (14)	0.0046 (13)	−0.0097 (11)	−0.0012 (14)
C18	0.0688 (17)	0.076 (2)	0.0592 (16)	0.0125 (18)	0.0086 (13)	0.0127 (16)
C19	0.067 (2)	0.067 (2)	0.099 (3)	0.0136 (19)	0.0331 (19)	0.006 (2)
C20	0.0450 (12)	0.0463 (16)	0.0586 (16)	0.0016 (12)	−0.0012 (11)	0.0023 (13)
C21	0.0546 (14)	0.0414 (15)	0.0601 (16)	−0.0046 (13)	−0.0038 (12)	−0.0011 (13)
C22	0.0543 (14)	0.0408 (15)	0.0597 (14)	−0.0028 (13)	0.0054 (11)	−0.0013 (13)
C23	0.0528 (14)	0.0448 (17)	0.0614 (16)	0.0028 (13)	0.0059 (12)	−0.0001 (13)
C24	0.0641 (16)	0.0458 (17)	0.0671 (17)	0.0049 (15)	0.0159 (13)	0.0026 (14)
C25	0.097 (3)	0.056 (2)	0.158 (4)	0.024 (2)	0.041 (3)	0.012 (3)
C26	0.0605 (19)	0.081 (3)	0.112 (3)	0.0096 (19)	0.0097 (17)	0.012 (2)

### Geometric parameters (Å, º)

**Table:**

O1—C3	1.432 (4)	C10—C19	1.489 (5)
O1—H1A	0.8200	C11—C12	1.525 (4)
O2—C5	1.440 (4)	C11—H11A	0.9700
O2—H2A	0.8200	C11—H11B	0.9700
O3—C19	1.127 (7)	C12—C13	1.528 (4)
O3A—C19	1.169 (10)	C12—H12A	0.9700
O4—C21	1.343 (4)	C12—H12B	0.9700
O4—C14	1.473 (3)	C13—C18	1.517 (5)
O5—C24	1.219 (4)	C13—C17	1.543 (4)
N1—C24	1.357 (4)	C13—C14	1.544 (4)
N1—C25	1.439 (5)	C14—C15	1.548 (4)
N1—C26	1.455 (5)	C15—C16	1.540 (5)
C1—C2	1.511 (5)	C15—H15A	0.9700
C1—C10	1.538 (5)	C15—H15B	0.9700
C1—H1B	0.9700	C16—C17	1.541 (5)
C1—H1C	0.9700	C16—H16A	0.9700
C2—C3	1.500 (5)	C16—H16B	0.9700
C2—H2B	0.9700	C17—C20	1.506 (4)
C2—H2C	0.9700	C17—H17A	0.9800
C3—C4	1.526 (5)	C18—H18A	0.9600
C3—H3A	0.9800	C18—H18B	0.9600
C4—C5	1.517 (5)	C18—H18C	0.9600
C4—H4A	0.9700	C19—H19A	0.9300
C4—H4B	0.9700	C19—H19B	0.9300
C5—C6	1.519 (4)	C20—C21	1.342 (4)
C5—C10	1.572 (4)	C20—C22	1.441 (4)
C6—C7	1.518 (5)	C21—H21A	0.9300
C6—H6A	0.9700	C22—C23	1.331 (4)
C6—H6B	0.9700	C22—H22A	0.9300
C7—C8	1.534 (4)	C23—C24	1.477 (4)
C7—H7A	0.9700	C23—H23A	0.9300
C7—H7B	0.9700	C25—H25A	0.9600
C8—C14	1.515 (4)	C25—H25B	0.9600
C8—C9	1.540 (4)	C25—H25C	0.9600
C8—H8A	0.9800	C26—H26A	0.9600
C9—C11	1.535 (4)	C26—H26B	0.9600
C9—C10	1.560 (4)	C26—H26C	0.9600
C9—H9A	0.9800		
			
C3—O1—H1A	109.5	C13—C12—H12B	108.9
C5—O2—H2A	109.5	H12A—C12—H12B	107.7
C21—O4—C14	115.3 (2)	C18—C13—C12	109.6 (3)
C24—N1—C25	118.9 (3)	C18—C13—C17	113.0 (3)
C24—N1—C26	124.9 (3)	C12—C13—C17	113.0 (2)
C25—N1—C26	116.1 (3)	C18—C13—C14	114.3 (2)
C2—C1—C10	114.3 (3)	C12—C13—C14	108.1 (2)
C2—C1—H1B	108.7	C17—C13—C14	98.5 (2)
C10—C1—H1B	108.7	O4—C14—C8	104.82 (18)
C2—C1—H1C	108.7	O4—C14—C13	108.3 (2)
C10—C1—H1C	108.7	C8—C14—C13	115.1 (2)
H1B—C1—H1C	107.6	O4—C14—C15	107.6 (2)
C3—C2—C1	111.8 (3)	C8—C14—C15	116.2 (3)
C3—C2—H2B	109.3	C13—C14—C15	104.5 (2)
C1—C2—H2B	109.3	C16—C15—C14	106.0 (2)
C3—C2—H2C	109.3	C16—C15—H15A	110.5
C1—C2—H2C	109.3	C14—C15—H15A	110.5
H2B—C2—H2C	107.9	C16—C15—H15B	110.5
O1—C3—C2	111.6 (3)	C14—C15—H15B	110.5
O1—C3—C4	107.9 (3)	H15A—C15—H15B	108.7
C2—C3—C4	109.7 (3)	C15—C16—C17	103.4 (2)
O1—C3—H3A	109.2	C15—C16—H16A	111.1
C2—C3—H3A	109.2	C17—C16—H16A	111.1
C4—C3—H3A	109.2	C15—C16—H16B	111.1
C5—C4—C3	114.7 (3)	C17—C16—H16B	111.1
C5—C4—H4A	108.6	H16A—C16—H16B	109.0
C3—C4—H4A	108.6	C20—C17—C16	108.3 (2)
C5—C4—H4B	108.6	C20—C17—C13	107.9 (2)
C3—C4—H4B	108.6	C16—C17—C13	103.5 (3)
H4A—C4—H4B	107.6	C20—C17—H17A	112.2
O2—C5—C4	110.1 (3)	C16—C17—H17A	112.2
O2—C5—C6	105.8 (2)	C13—C17—H17A	112.2
C4—C5—C6	110.6 (3)	C13—C18—H18A	109.5
O2—C5—C10	108.2 (2)	C13—C18—H18B	109.5
C4—C5—C10	110.8 (2)	H18A—C18—H18B	109.5
C6—C5—C10	111.2 (3)	C13—C18—H18C	109.5
C7—C6—C5	113.8 (2)	H18A—C18—H18C	109.5
C7—C6—H6A	108.8	H18B—C18—H18C	109.5
C5—C6—H6A	108.8	O3—C19—O3A	83.9 (6)
C7—C6—H6B	108.8	O3—C19—C10	135.9 (5)
C5—C6—H6B	108.8	O3A—C19—C10	126.5 (6)
H6A—C6—H6B	107.7	O3—C19—H19A	112.1
C6—C7—C8	111.5 (3)	O3A—C19—H19A	49.4
C6—C7—H7A	109.3	C10—C19—H19A	112.1
C8—C7—H7A	109.3	O3—C19—H19B	47.2
C6—C7—H7B	109.3	O3A—C19—H19B	116.8
C8—C7—H7B	109.3	C10—C19—H19B	116.8
H7A—C7—H7B	108.0	H19A—C19—H19B	107.7
C14—C8—C7	111.8 (2)	C21—C20—C22	118.8 (3)
C14—C8—C9	110.88 (19)	C21—C20—C17	117.7 (3)
C7—C8—C9	111.6 (2)	C22—C20—C17	123.4 (2)
C14—C8—H8A	107.4	C20—C21—O4	125.0 (3)
C7—C8—H8A	107.4	C20—C21—H21A	117.5
C9—C8—H8A	107.4	O4—C21—H21A	117.5
C11—C9—C8	110.4 (2)	C23—C22—C20	127.3 (3)
C11—C9—C10	113.1 (2)	C23—C22—H22A	116.4
C8—C9—C10	112.5 (2)	C20—C22—H22A	116.4
C11—C9—H9A	106.8	C22—C23—C24	120.8 (3)
C8—C9—H9A	106.8	C22—C23—H23A	119.6
C10—C9—H9A	106.8	C24—C23—H23A	119.6
C19—C10—C1	106.9 (3)	O5—C24—N1	121.3 (3)
C19—C10—C9	111.3 (3)	O5—C24—C23	121.3 (3)
C1—C10—C9	112.6 (2)	N1—C24—C23	117.5 (3)
C19—C10—C5	107.4 (2)	N1—C25—H25A	109.5
C1—C10—C5	107.8 (3)	N1—C25—H25B	109.5
C9—C10—C5	110.6 (2)	H25A—C25—H25B	109.5
C12—C11—C9	111.5 (2)	N1—C25—H25C	109.5
C12—C11—H11A	109.3	H25A—C25—H25C	109.5
C9—C11—H11A	109.3	H25B—C25—H25C	109.5
C12—C11—H11B	109.3	N1—C26—H26A	109.5
C9—C11—H11B	109.3	N1—C26—H26B	109.5
H11A—C11—H11B	108.0	H26A—C26—H26B	109.5
C11—C12—C13	113.2 (2)	N1—C26—H26C	109.5
C11—C12—H12A	108.9	H26A—C26—H26C	109.5
C13—C12—H12A	108.9	H26B—C26—H26C	109.5
C11—C12—H12B	108.9		
			
C10—C1—C2—C3	57.5 (4)	C7—C8—C14—C13	179.7 (2)
C1—C2—C3—O1	65.9 (4)	C9—C8—C14—C13	54.5 (3)
C1—C2—C3—C4	−53.7 (4)	C7—C8—C14—C15	57.1 (3)
O1—C3—C4—C5	−67.4 (4)	C9—C8—C14—C15	−68.1 (3)
C2—C3—C4—C5	54.5 (4)	C18—C13—C14—O4	−47.9 (3)
C3—C4—C5—O2	65.0 (3)	C12—C13—C14—O4	−170.2 (2)
C3—C4—C5—C6	−178.4 (3)	C17—C13—C14—O4	72.2 (2)
C3—C4—C5—C10	−54.7 (4)	C18—C13—C14—C8	69.0 (4)
O2—C5—C6—C7	−171.6 (3)	C12—C13—C14—C8	−53.3 (3)
C4—C5—C6—C7	69.2 (4)	C17—C13—C14—C8	−170.9 (2)
C10—C5—C6—C7	−54.3 (4)	C18—C13—C14—C15	−162.4 (3)
C5—C6—C7—C8	55.2 (4)	C12—C13—C14—C15	75.3 (3)
C6—C7—C8—C14	−178.7 (3)	C17—C13—C14—C15	−42.3 (3)
C6—C7—C8—C9	−53.8 (3)	O4—C14—C15—C16	−93.9 (3)
C14—C8—C9—C11	−53.4 (3)	C8—C14—C15—C16	149.1 (2)
C7—C8—C9—C11	−178.7 (3)	C13—C14—C15—C16	21.1 (3)
C14—C8—C9—C10	179.2 (2)	C14—C15—C16—C17	9.2 (3)
C7—C8—C9—C10	53.9 (3)	C15—C16—C17—C20	78.0 (3)
C2—C1—C10—C19	−170.2 (3)	C15—C16—C17—C13	−36.4 (3)
C2—C1—C10—C9	67.3 (4)	C18—C13—C17—C20	55.1 (3)
C2—C1—C10—C5	−55.0 (3)	C12—C13—C17—C20	−179.8 (2)
C11—C9—C10—C19	−59.4 (3)	C14—C13—C17—C20	−66.0 (3)
C8—C9—C10—C19	66.6 (3)	C18—C13—C17—C16	169.7 (2)
C11—C9—C10—C1	60.6 (3)	C12—C13—C17—C16	−65.2 (3)
C8—C9—C10—C1	−173.4 (2)	C14—C13—C17—C16	48.6 (3)
C11—C9—C10—C5	−178.6 (2)	C1—C10—C19—O3	−147.7 (7)
C8—C9—C10—C5	−52.7 (3)	C9—C10—C19—O3	−24.3 (8)
O2—C5—C10—C19	46.2 (3)	C5—C10—C19—O3	96.8 (7)
C4—C5—C10—C19	167.0 (3)	C1—C10—C19—O3A	−22.8 (8)
C6—C5—C10—C19	−69.6 (4)	C9—C10—C19—O3A	100.6 (7)
O2—C5—C10—C1	−68.6 (3)	C5—C10—C19—O3A	−138.3 (7)
C4—C5—C10—C1	52.2 (3)	C16—C17—C20—C21	−77.4 (3)
C6—C5—C10—C1	175.6 (2)	C13—C17—C20—C21	34.0 (4)
O2—C5—C10—C9	167.8 (2)	C16—C17—C20—C22	99.9 (3)
C4—C5—C10—C9	−71.4 (3)	C13—C17—C20—C22	−148.8 (3)
C6—C5—C10—C9	52.0 (3)	C22—C20—C21—O4	−177.6 (3)
C8—C9—C11—C12	55.2 (3)	C17—C20—C21—O4	−0.2 (5)
C10—C9—C11—C12	−177.7 (3)	C14—O4—C21—C20	5.0 (4)
C9—C11—C12—C13	−57.2 (4)	C21—C20—C22—C23	172.7 (3)
C11—C12—C13—C18	−71.4 (3)	C17—C20—C22—C23	−4.5 (5)
C11—C12—C13—C17	161.6 (3)	C20—C22—C23—C24	−179.5 (3)
C11—C12—C13—C14	53.8 (3)	C25—N1—C24—O5	−1.4 (5)
C21—O4—C14—C8	−166.9 (2)	C26—N1—C24—O5	173.2 (4)
C21—O4—C14—C13	−43.6 (3)	C25—N1—C24—C23	178.1 (4)
C21—O4—C14—C15	68.8 (3)	C26—N1—C24—C23	−7.3 (5)
C7—C8—C14—O4	−61.5 (3)	C22—C23—C24—O5	−24.2 (5)
C9—C8—C14—O4	173.3 (2)	C22—C23—C24—N1	156.4 (3)

### Hydrogen-bond geometry (Å, º)

**Table:**

*D*—H···*A*	*D*—H	H···*A*	*D*···*A*	*D*—H···*A*
O1—H1*A*···O5^i^	0.82	1.97	2.790 (3)	178
C25—H25*C*···O1^ii^	0.96	2.64	3.513 (3)	152

Symmetry codes: (i) *x*, *y*+1, *z*; (ii) *x*+1, *y*−1, *z*.
